# Circulating tumor DNA as a predictive biomarker for colorectal cancer postsurgical recurrence: a systematic review and meta-analysis

**DOI:** 10.1007/s12094-025-04073-y

**Published:** 2025-10-17

**Authors:** Atta Ullah Khan, Yasameen Hameed Jasim, Kanza Shahid, Jasur Saidov, Zamira Atamuratova, Dilbar Urazbaeva

**Affiliations:** 1 Department of Precision Medicine, University of Campania “Luigi Vanvitelli”, 80138 Naples, Italy; 2 Department of Applied Chemistry, College of Applied Sciences, Samarra University, Samarra, Iraq; 3 Department of Biomedicine, Atta Ur Rahman School of Applied Biosciences, National University of Science and Technology (NUST), Islamabad, Pakistan; 4 Department of Medicine, Termez University of Economics and Service, Termez, Uzbekistan; 5 Department of Pedagogy and Psychology, Urgench State University, Urgench, Uzbekistan; 6 Department of Psychology and Medicine, Mamun University, Khiva, Uzbekistan

**Keywords:** CtDNA, Prognostic biomarker, Recurrence, Postsurgical, Colorectal cancer

## Abstract

**Purpose:**

Colorectal carcinoma constitutes a predominant etiology of oncological mortality globally. This systematic review and meta-analysis elucidated the prognostic utility of circulating tumor DNA (ctDNA) as a predictive biomarker for postsurgical recurrence in colorectal cancer patients.

**Methods:**

Two independent investigators conducted systematic literature search across PubMed, Web of Science, Embase, Scopus, and clinical trial registries. Studies investigating ctDNA prognostic significance for colorectal cancer recurrence were incorporated. Random-effects models were implemented utilizing restricted maximum likelihood methodology. The study quality was assessed using Newcastle–Ottawa Scale.

**Results:**

Following screening of 2259 records, 11 studies were incorporated. ctDNA-positive patients exhibited significantly elevated recurrence risk as compared to ctDNA-negative counterparts (pooled HR: 2.34; 95% CI: 1.90–2.79; *p* < 0.001). Moderate heterogeneity was observed (*I*^2^ = 66.40%), attributable to patient stage distribution, sampling timing, detection platforms, and mutational panel variations. Stage I-III patients demonstrated exceptional consistency (HR: 2.04, *I*^2^ = 0.00%). Detection platforms showed robust performance: droplet digital PCR (HR: 3.63), next-generation sequencing (HR: 2.67), and Safe-SeqS (HR: 2.16), with no significant differences (*p* = 0.10). Adjuvant chemotherapy analysis revealed differential performance: treated patients (HR: 2.50; 95% CI: 2.08–2.93) versus untreated (HR: 1.70; 95% CI: 1.07–2.34; *p* = 0.04). Extended analysis confirmed prognostic utility for overall survival (HR: 2.24) and surveillance recurrence-free survival (HR: 3.54).

**Conclusions:**

ctDNA represents a robust prognostic biomarker for postsurgical colorectal cancer recurrence with consistent cross-platform performance. Enhanced prognostic value in adjuvant chemotherapy patients supports personalized surveillance implementation, though methodological standardization remains warranted.

**Supplementary Information:**

The online version contains supplementary material available at 10.1007/s12094-025-04073-y.

## Introduction

Colorectal cancer (CRC) remains a major global health challenge, ranking among the leading causes of cancer-related deaths worldwide, with over 1.9 million new cases and 935,000 deaths estimated in 2020 according to GLOBOCAN data [1, 2]. Despite advances in surgical and systemic treatments, disease recurrence after curative-intent therapy continues to be a significant obstacle, affecting approximately one-third of patients who achieve no detectable tumor at initial evaluation [3, 4]. Notably, most recurrences emerge within the first two years post-treatment, underscoring the likelihood of occult residual disease or micro-metastases that evade standard diagnostic modalities [3, 5].

The prognosis of CRC varies dramatically by stage at diagnosis. Although early-stage disease (stage I) boasts a favorable 5 year survival rate exceeding 90%, survival rates plummet to below 15% in patients with metastatic (stage IV) CRC [6, 7]. Traditional staging, which evaluates tumor invasion depth, lymph node involvement, and distant metastasis, is instrumental in initial treatment planning [8]. However, its predictive accuracy is limited for intermediate stages (II and III), where deciding on adjuvant therapy and surveillance strategies remains challenging [7, 9]. Consequently, vigilant follow-up is necessary to detect asymptomatic recurrence early enough for effective intervention [4, 10].

Currently, the biomarker carcinoembryonic antigen (CEA) is the only widely endorsed serum marker for postoperative monitoring. However, its limited sensitivity (40–70%) and specificity (80–90%) restrict its usefulness in reliably identifying recurrence at an early, treatable stage [11–13]. This limitation has driven intensive research into novel biomarkers that can enhance prognostic precision and enable real-time surveillance [14].

In the recent years, circulating tumor DNA (ctDNA), representing tumor-derived genetic fragments in the bloodstream, has emerged as a promising biomarker for monitoring CRC [15–17]. By detecting tumor-specific mutations through highly sensitive molecular assays, ctDNA analysis holds potential for identifying minimal residual disease and predicting recurrence risk more accurately than traditional methods [15, 18, 19]. The biological mechanism underlying ctDNA release involves multiple pathways including apoptosis, necrosis, and active secretion from tumor cells, making it a real-time indicator of tumor burden [16, 20]. Nonetheless, the heterogeneity of somatic mutations across tumors and the evolving genetic landscape during cancer progression pose challenges to broad application [21, 22].

Given the growing volume of research evaluating ctDNA in CRC, a comprehensive synthesis of available evidence is essential to establish its clinical utility. This systematic review and meta-analysis aim to critically assess the prognostic value of ctDNA detection for predicting recurrence in CRC patients post-treatment, providing evidence-based insights to guide future clinical practice and research.

## Methods

### Literature search strategy

A comprehensive literature search was conducted in major electronic databases, including PubMed, Embase, Web of Science, and The Cochrane Library, to identify studies evaluating the association of postoperative ctDNA with colorectal cancer recurrence. The search included studies published up to August 2025 without language restrictions. Search terms combined controlled vocabulary and free-text terms related to "circulating tumor DNA," "ctDNA," "colorectal cancer," "postoperative," "recurrence," and "prognosis". Bibliographies of relevant articles and reviews were also screened for additional eligible studies. The search strategy followed established guidelines for systematic reviews [23]. The protocol for this systematic review and meta-analysis was prospectively registered (CRD420251138539) in the PROSPERO database following established guidelines for systematic review protocols. For detailed search strategy refer to Online Resource 1.

### Inclusion and exclusion criteria

Eligible studies met the following criteria: (1) involved patients with histologically confirmed colorectal cancer who underwent curative-intent surgery; (2) assessed postoperative ctDNA status as a biomarker; (3) reported recurrence outcomes, including recurrence-free survival (RFS), disease-free survival (DFS) for recurrence according to ctDNA status; (4) provided hazard ratios (HRs), or sufficient raw data for effect size estimation; (5) were cohort studies, case–control studies, or clinical trials.

Studies were excluded based on the following criteria: (1) studies not focused on prognosis or recurrence biomarker applications; (2) studies with incomplete outcome data or insufficient statistical information for meta-analysis; (3) duplicate cohort reports using the same patient population; (4) non-English language studies; (5) case reports, editorials, reviews, and conference abstracts without peer-reviewed full-text publication; (6) studies focusing solely on therapeutic monitoring without prognostic outcomes; and (7) studies including only metastatic patients without surgical resection.

### Data extraction and quality assessment

Two reviewers independently extracted data using a standardized form, including study characteristics (publication year, country, sample size), patient demographics, ctDNA assay methods, recurrence outcomes, and effect estimates with 95% confidence intervals (CIs). Discrepancies were resolved by consensus or consultation with a third reviewer. The methodological quality of included studies was assessed using the Newcastle–Ottawa Scale (NOS) for observational studies [24, 25]. The scale evaluates three domains: selection of study groups (4 points), comparability of groups (2 points), and assessment of outcomes (3 points), with a maximum score of 9 points. Studies scoring ≥ 7 points were considered high quality, 5–6 points moderate quality, and < 5 points low quality. Assessment was performed independently by two investigators, with disagreements resolved through discussion and consensus, or consultation with a third investigator when necessary.

### Statistical analysis

Meta-analyses were performed to pool HRs for recurrence-free survival comparing ctDNA-positive versus ctDNA-negative patients post-surgery. Random-effects models were applied due to anticipated clinical and methodological heterogeneity across studies [26]. Heterogeneity was quantified using the *I*^2^ statistic and Cochran's *Q* test [27]. Subgroup analyses were conducted by ctDNA assay type, Adjuvant Chemotherapy, and CRC stage when data allowed. Publication bias was evaluated by funnel plot inspection and Egger's test [28]. All analyses were performed using Stata (version 17) software.

## Results

### Study characteristics and selection process

The comprehensive systematic literature search identified 2259 bibliographic records from multiple databases and registries, encompassing PubMed (*n* = 330), Web of Science (*n* = 360), Embase (*n* = 460), Scopus (*n* = 920), manual bibliographic searches (*n* = 139), and clinical trial registries including ClinicalTrials.gov (*n* = 39), EU Clinical Trials Register (*n* = 8), and Japan Primary Registries Network (*n* = 3). Following the elimination of 900 duplicate records, 1359 citations underwent systematic title and abstract screening. Subsequently, 1200 records were excluded based on predetermined criteria, with 159 reports retrieved for comprehensive full-text evaluation.

**Fig. 1:**
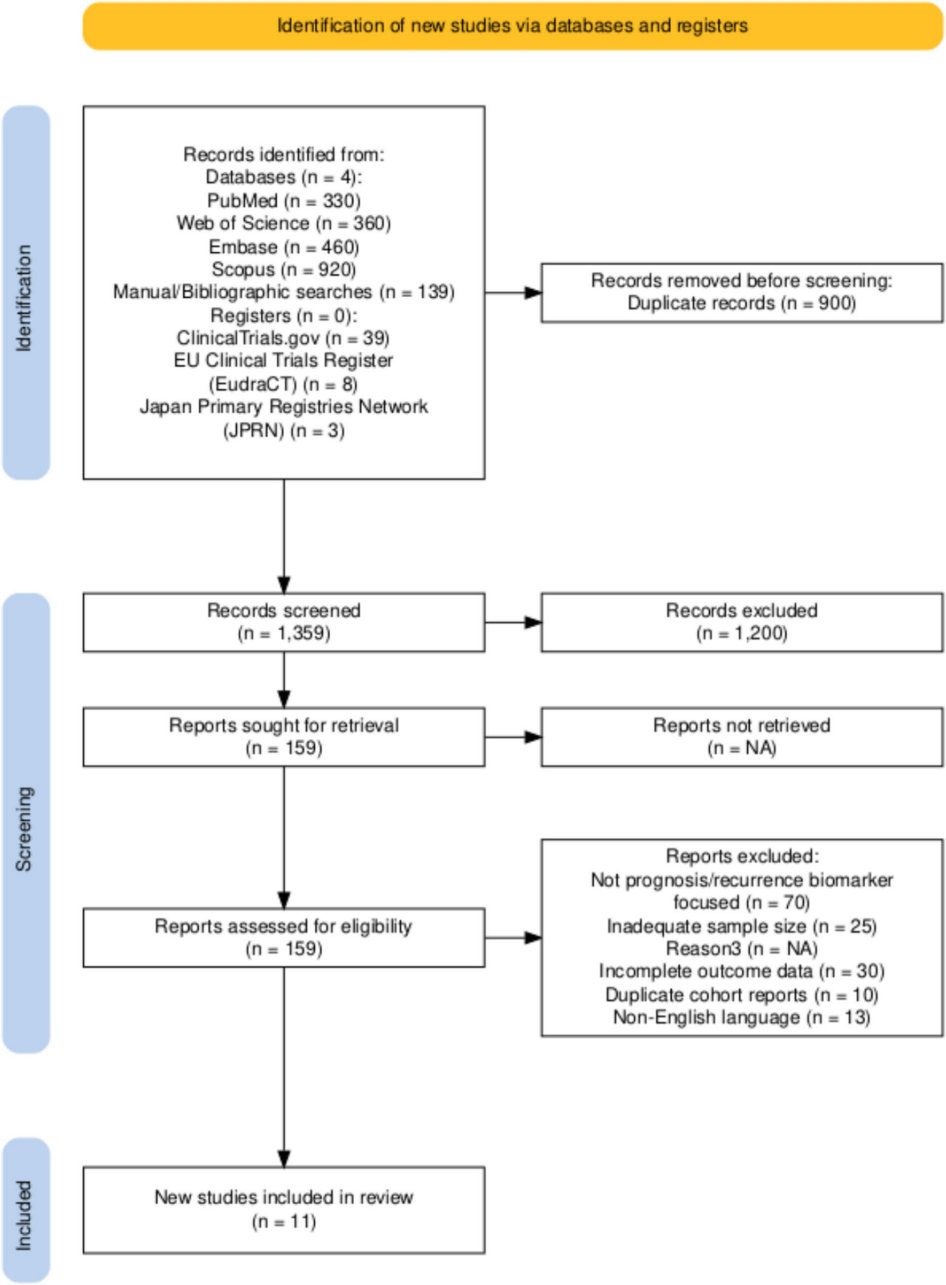
PRISMA flow chart

A total of 148 reports were systematically excluded based on rigorous eligibility criteria: 70 studies lacked focus on prognostic/recurrence biomarkers, 25 demonstrated inadequate sample sizes for meaningful statistical analysis, 30 exhibited incomplete outcome data precluding meta-analytic synthesis, 10 represented duplicate cohort reports, and 13 were published in non-English languages. Consequently, 11 studies satisfied the predetermined inclusion criteria and were incorporated into the quantitative synthesis (Fig. 1) [29–39]. For detailed characteristics refer to (Online resource 2).

The incorporated studies encompassed heterogeneous patient populations and diverse methodological approaches. Studies employed various ctDNA detection methodologies including next-generation sequencing (NGS), Safe-SeqS, droplet digital PCR (ddPCR), quantitative PCR (qPCR), and alternative technological platforms. The cancer staging encompassed various disease stages, with studies incorporating different combinations of stage I–IV patients.

### Study quality assessment

The methodological quality of included studies was systematically evaluated using the Newcastle–Ottawa Scale (NOS) for observational studies. Overall NOS scores ranged from 6 to 9 stars (median: 8 stars), with ten studies (90.9%) achieving high-quality ratings (≥ 7 stars) and one study receiving a moderate-quality rating (6 stars). In the selection domain, all studies achieved maximum scores for adequate case definition and representativeness. The comparability domain showed variation, with seven studies (63.6%) receiving maximum scores for controlling important confounding factors. Outcome assessment was consistently robust, with ten studies (90.9%) achieving maximum scores for adequate follow-up duration and completeness. The predominantly high NOS scores support the reliability of the synthesized evidence and strengthen confidence in the meta-analytic findings (Online Resource 3).

### ctDNA detection and colorectal cancer recurrence

**Fig. 2:**
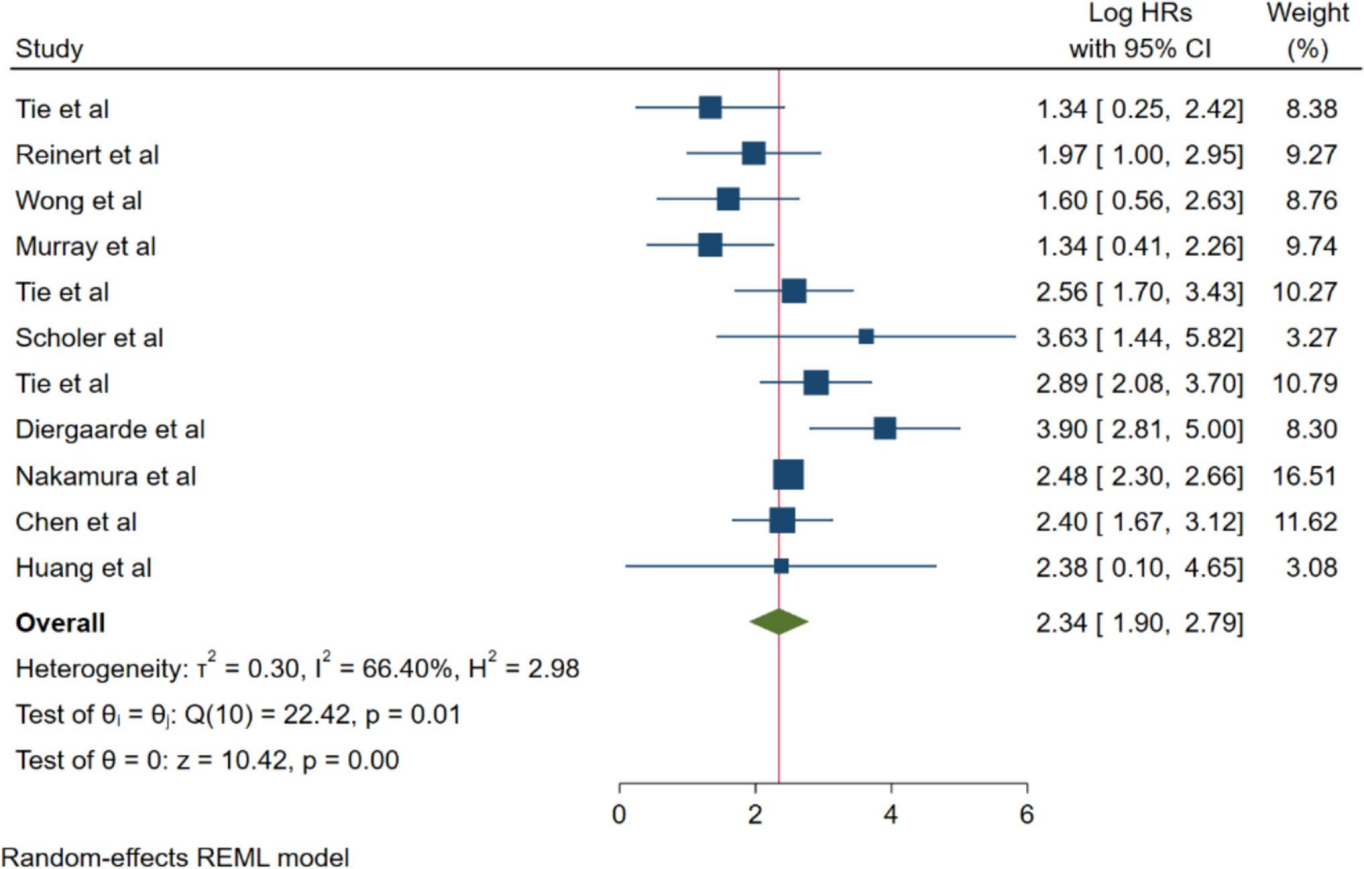
Forest plot for recurrence and ctDNA positivity

The primary meta-analytic synthesis of 11 studies demonstrated a statistically significant association between ctDNA positivity and elevated colorectal cancer recurrence risk. The pooled hazard ratio was 2.34 (95% CI: 1.90–2.79; *p* < 0.001), indicating that patients harboring detectable ctDNA exhibited more than double the recurrence risk as compared to those with undetectable ctDNA levels (Fig. 2).

Heterogeneity assessment revealed moderate inter-study statistical heterogeneity (*I*^2^ = 66.40%, *τ*^2^ = 0.30, *H*^2^ = 2.98; Q (10) = 22.42, *p* = 0.01). Despite this heterogeneity, the random-effects model provided robust effect estimates, with all individual studies consistently demonstrating hazard ratios exceeding unity, corroborating the prognostic utility of ctDNA across diverse study populations and methodological frameworks.

Individual study hazard ratios demonstrated considerable variability, ranging from 1.34 (95% CI: 0.25–2.42) [29], to 3.90 (95% CI: 2.81–5.00) [36], with the majority of investigations demonstrating statistically significant associations. Additional noteworthy individual study findings included Reinert et al. (HR: 1.97; 95% CI: 1.00–2.95) [30], Wong et al. (HR: 1.60; 95% CI: 0.56–2.63) [31], Murray et al. (HR: 1.34; 95% CI: 0.41–2.26) [32], Tie et al. (HR: 2.56; 95% CI: 1.70–3.43) [33], Scholer et al. (HR: 3.63; 95% CI: 1.44–5.82) [34], Tie et al. (HR: 2.89; 95% CI: 2.08–3.70) [35], Nakamura et al. (HR: 2.48; 95% CI: 2.30–2.66) [38], Chen et al. (HR: 2.40; 95% CI: 1.67–3.12) [37], and Huang et al. (HR: 2.38; 95% CI: 0.10–4.65) [39].

### Exploratory analysis

#### ctDNA and surveillance recurrence-free survival

**Fig. 3:**
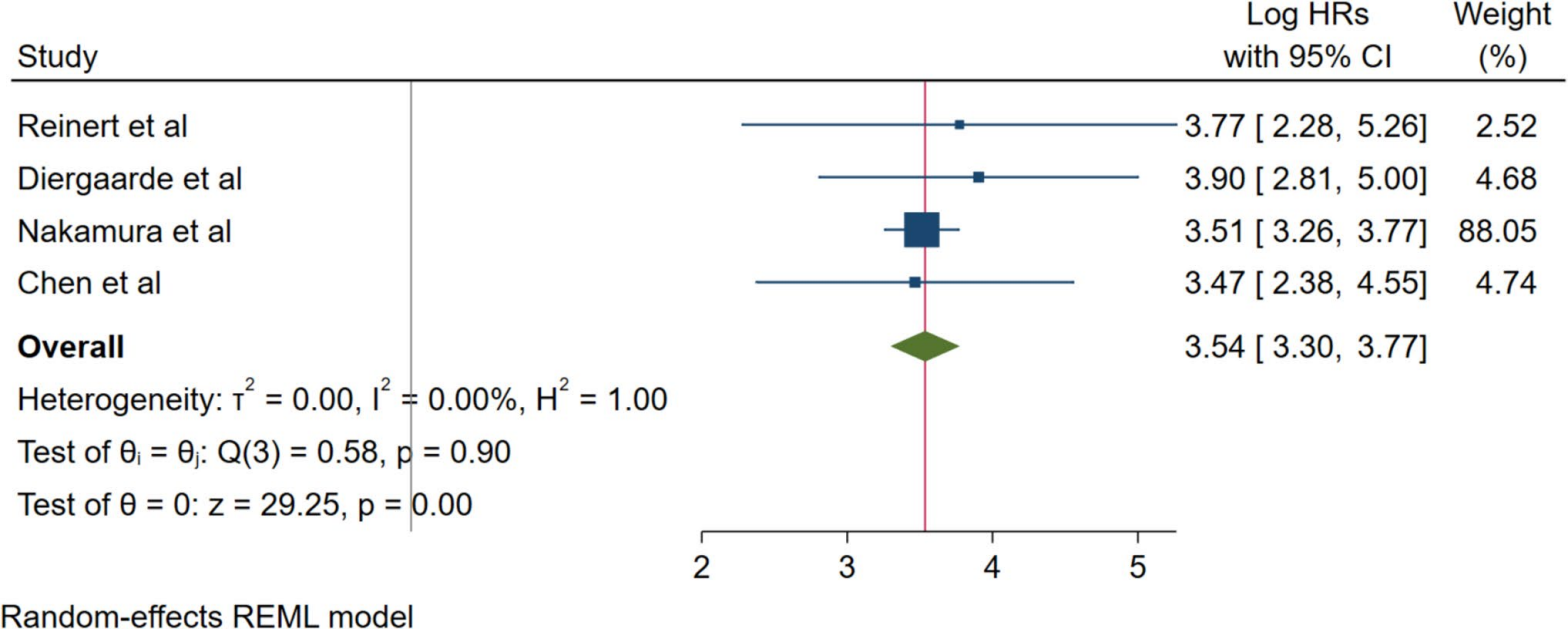
Forest plot for surveillance RFS and ctDNA positivity

A subset analysis of studies specifically reporting surveillance recurrence-free survival outcomes demonstrated exceptional prognostic performance of ctDNA. Four studies (Reinert et al., Diergaarde et al., Nakamura et al., and Chen et al.) contributed to this analysis, encompassing patients under active surveillance protocols. The pooled hazard ratio was 3.54 (95% CI: 3.30–3.77; *p* < 0.001), indicating that ctDNA-positive patients had more than three-fold increased risk of recurrence during surveillance as compared to ctDNA-negative patients. Notably, this analysis demonstrated exceptional homogeneity with no statistical heterogeneity observed (*I*^2^ = 0.00%, *τ*^2^ = 0.00, *H*^2^ = 1.00; Q (3) = 0.58, *p* = 0.90), suggesting consistent prognostic utility across different surveillance protocols and patient populations (Fig. 3).

#### ctDNA and overall survival

**Fig. 4:**
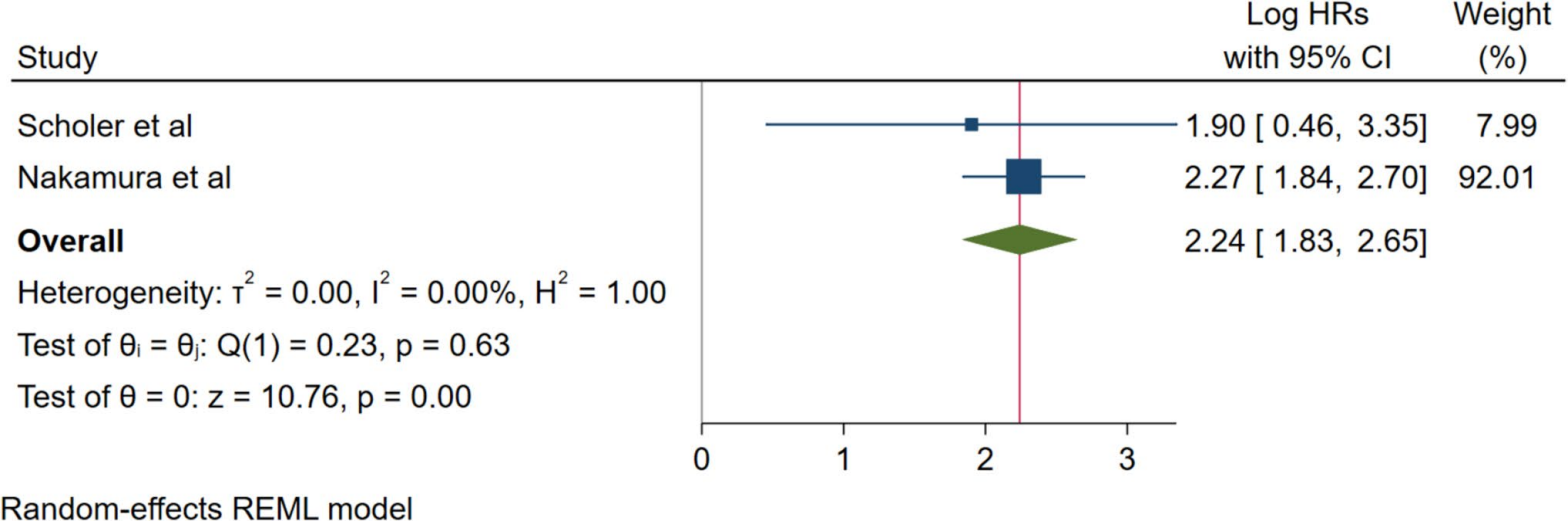
Forest plot for overall survival and ctDNA positivity

The prognostic utility of ctDNA extended beyond recurrence prediction to overall survival outcomes. Two studies (Scholer et al. and Nakamura et al.) provided data for overall survival analysis, demonstrating a pooled hazard ratio of 2.24 (95% CI: 1.83–2.65; *p* < 0.001). This indicates that ctDNA-positive patients exhibited more than double the mortality risk as compared to ctDNA-negative counterparts. The analysis showed perfect homogeneity with no heterogeneity observed (*I*^2^ = 0.00%, *τ*^2^ = 0.00, *H*^2^ = 1.00; Q (1) = 0.23, *p* = 0.63), supporting the consistent prognostic value of ctDNA for survival outcomes across different study populations and methodological approaches **(**Fig. 4).

### Subgroup stratification and heterogeneity exploration

#### Cancer stage stratification analysis

**Fig. 5:**
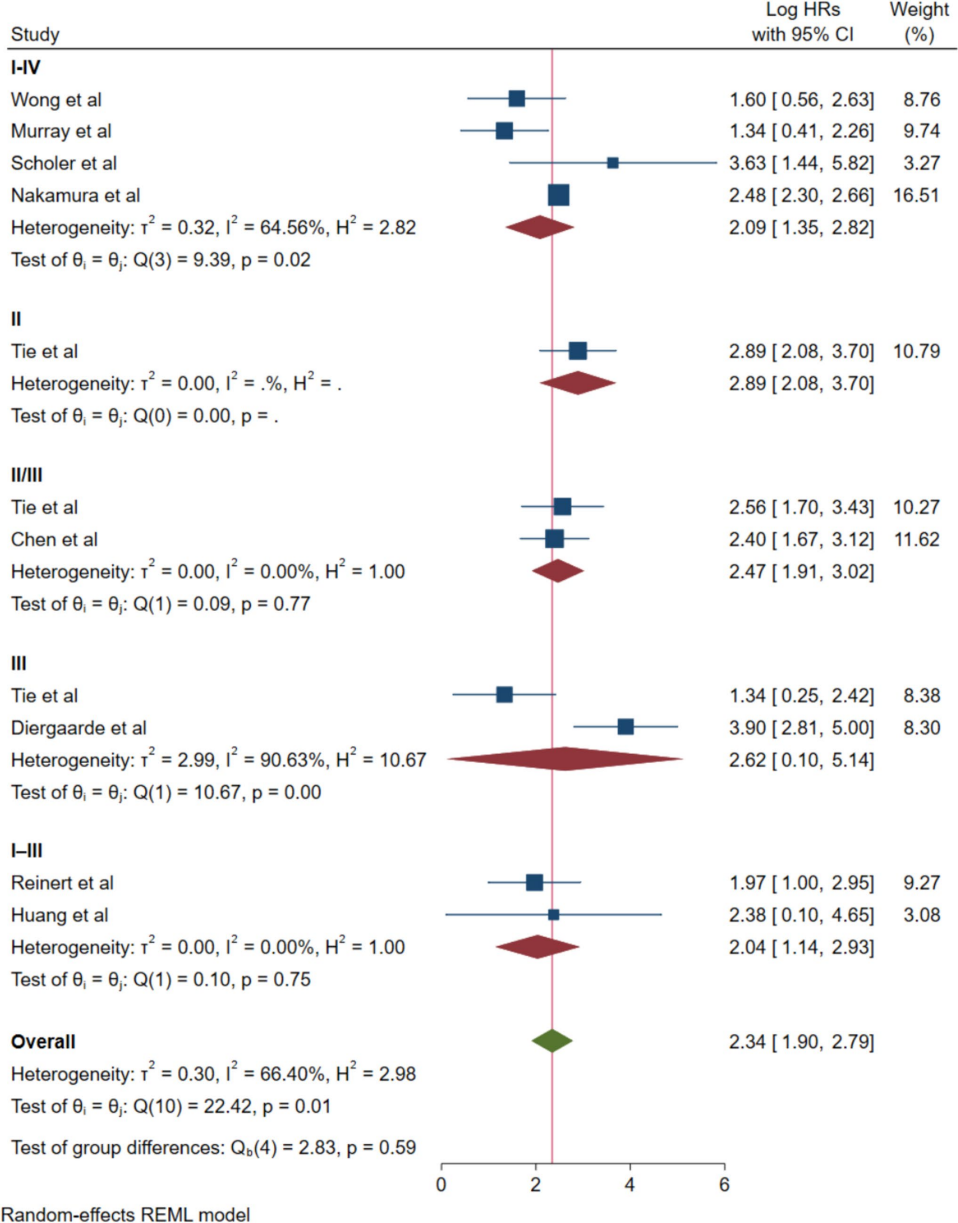
Forest plot for subgroup analysis by cancer stage

Subgroup stratification by cancer staging revealed pivotal insights regarding ctDNA prognostic utility across diverse disease stages (Fig. 5). Studies incorporating stage I-IV patients demonstrated a pooled HR of 2.09 (95% CI: 1.35–2.82) with moderate heterogeneity (*I*^2^ = 64.56%). This subgroup encompassed investigations by Wong et al.[31], Murray et al.[32], Scholer et al.[34], and Nakamura et al.[38].

Stage II patients demonstrated an HR of 2.89 (95% CI: 2.08–3.70) with negligible heterogeneity observed. Studies focusing on stage II/III patients exhibited consistent findings, while stage III patients demonstrated an HR of 2.62 (95% CI: 0.10–5.14), albeit with substantial heterogeneity (*I*^2^ = 90.63%), incorporating studies by Tie et al. and Diergaarde et al. [29, 36].

Remarkably, studies concentrating on combined stage I–III patients exhibited exceptional consistency with an HR of 2.04 (95% CI: 1.14–2.93) and negligible heterogeneity (*I*^2^ = 0.00%), encompassing investigations by Reinert et al. and Huang et al. This finding suggests that ctDNA may demonstrate particular reliability as a prognostic biomarker in early-stage disease where curative surgical intervention constitutes the primary therapeutic objective.

#### Detection methodology stratification analysis

**Fig. 6:**
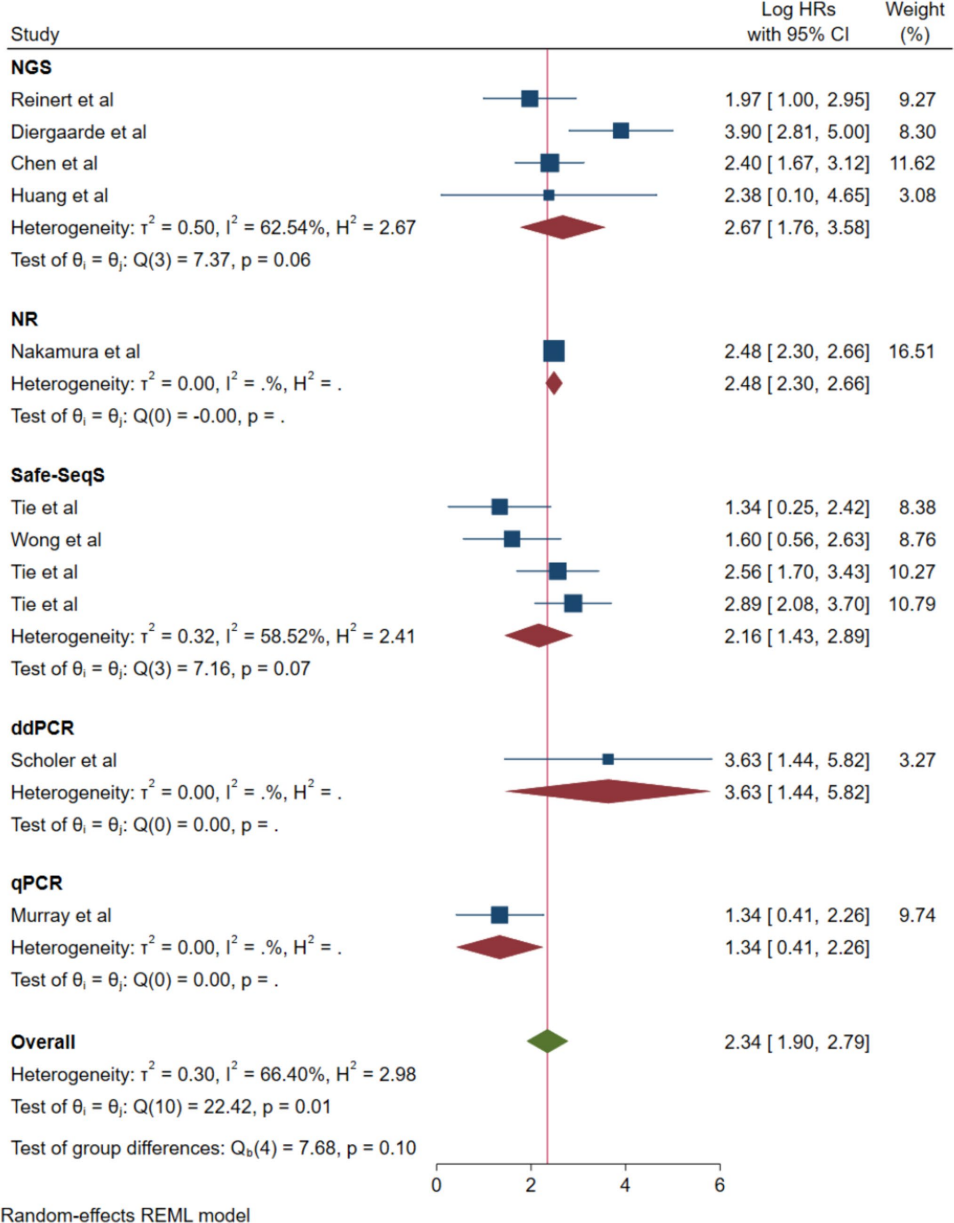
Forest plot for subgroup analysis by detection assay

Methodology-based subgroup stratification revealed differential performance characteristics across distinct ctDNA detection platforms (Fig. 6). Next-generation sequencing approaches demonstrated an HR of 2.67 (95% CI: 1.76–3.58) with moderate heterogeneity (*I*^2^ = 62.54%), incorporating studies by Reinert et al., Diergaarde et al., Chen et al., and Huang et al. [30, 36, 37, 39].

Safe-SeqS methodology exhibited an HR of 2.16 (95% CI: 1.43–2.89) with moderate heterogeneity (*I*^2^ = 58.52%), encompassing multiple investigations by Tie et al. Droplet digital PCR demonstrated substantial effect magnitude with an HR of 3.63 (95% CI: 1.44–5.82) and negligible heterogeneity, represented by the Scholer et al. investigation [34].

Quantitative PCR demonstrated an HR of 1.34 (95% CI: 0.41–2.26) with negligible heterogeneity, based on the Murray et al. study. The methodology designated as "NR" (not reported) exhibited an HR of 2.48 (95% CI: 2.30–2.66) with negligible heterogeneity, represented by the Nakamura et al. investigation [38].

The subgroup difference analysis demonstrated Q₍₄₎ = 7.68, *p* = 0.10, indicating absence of statistically significant differences between detection methodologies, although the varied effect magnitudes suggest potential methodological influences on ctDNA performance characteristics.

#### Adjuvant chemotherapy stratification analysis

**Fig. 7:**
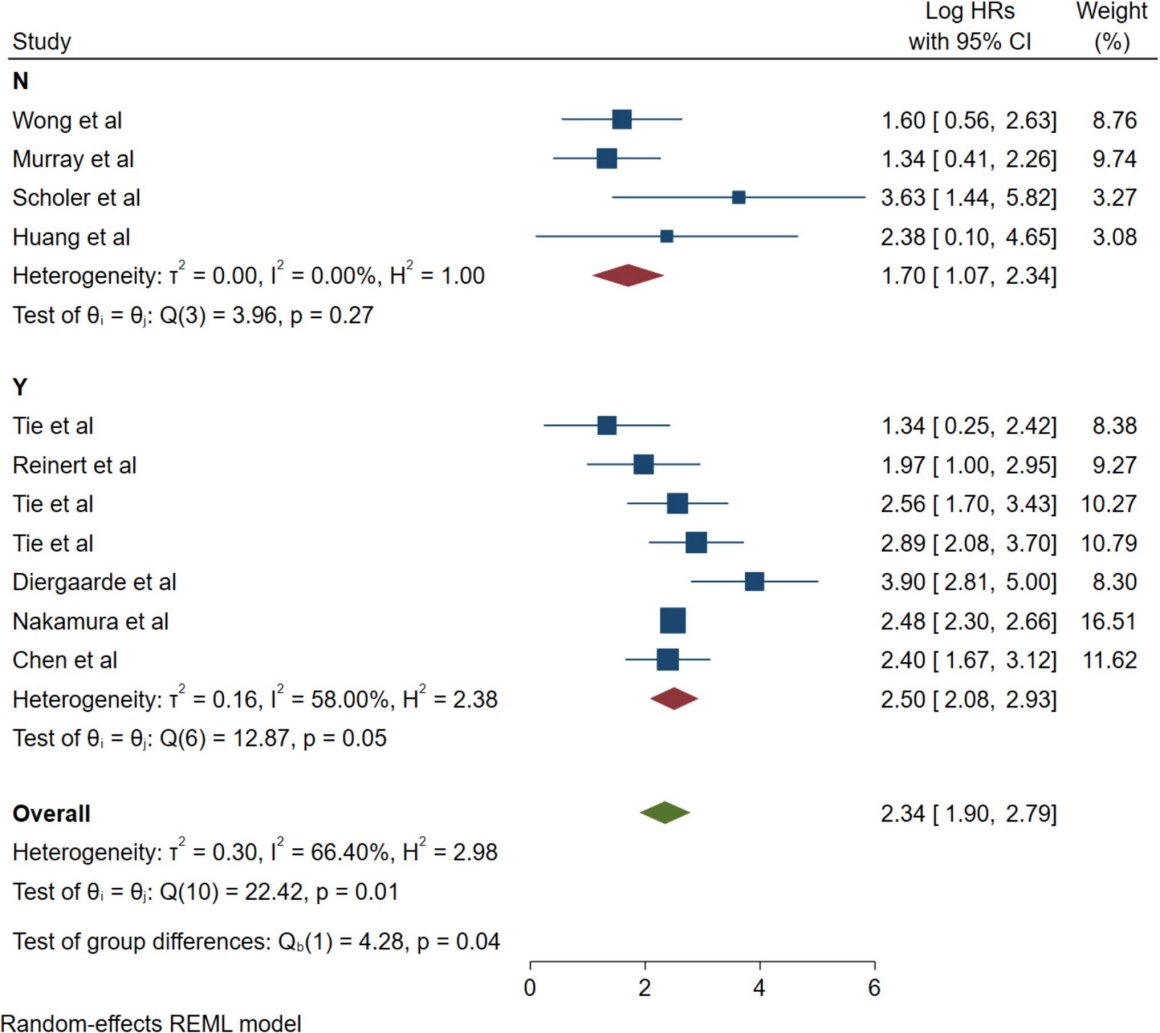
Forest plot for subgroup analysis by adjuvant chemotherapy

Subgroup stratification by adjuvant chemotherapy status demonstrated consistent ctDNA prognostic utility across treatment paradigms. Patients without adjuvant chemotherapy (N group, 4 studies) showed a pooled HR of 1.70 (95% CI: 1.07–2.34) with no heterogeneity (*I*^2^ = 0.00%, *p* = 0.27). Patients receiving adjuvant chemotherapy (Y group, 7 studies) demonstrated an HR of 2.50 (95% CI: 2.08–2.93) with moderate heterogeneity (*I*^2^ = 58.00%, *p* = 0.05). The test for subgroup differences was statistically significant (Q₀ (1) = 4.28, *p* = 0.04), indicating that ctDNA demonstrates stronger prognostic value in patients receiving adjuvant chemotherapy as compared to those without adjuvant treatment (Fig. 7).

### Publication bias and sensitivity analysis

**Fig. 8:**
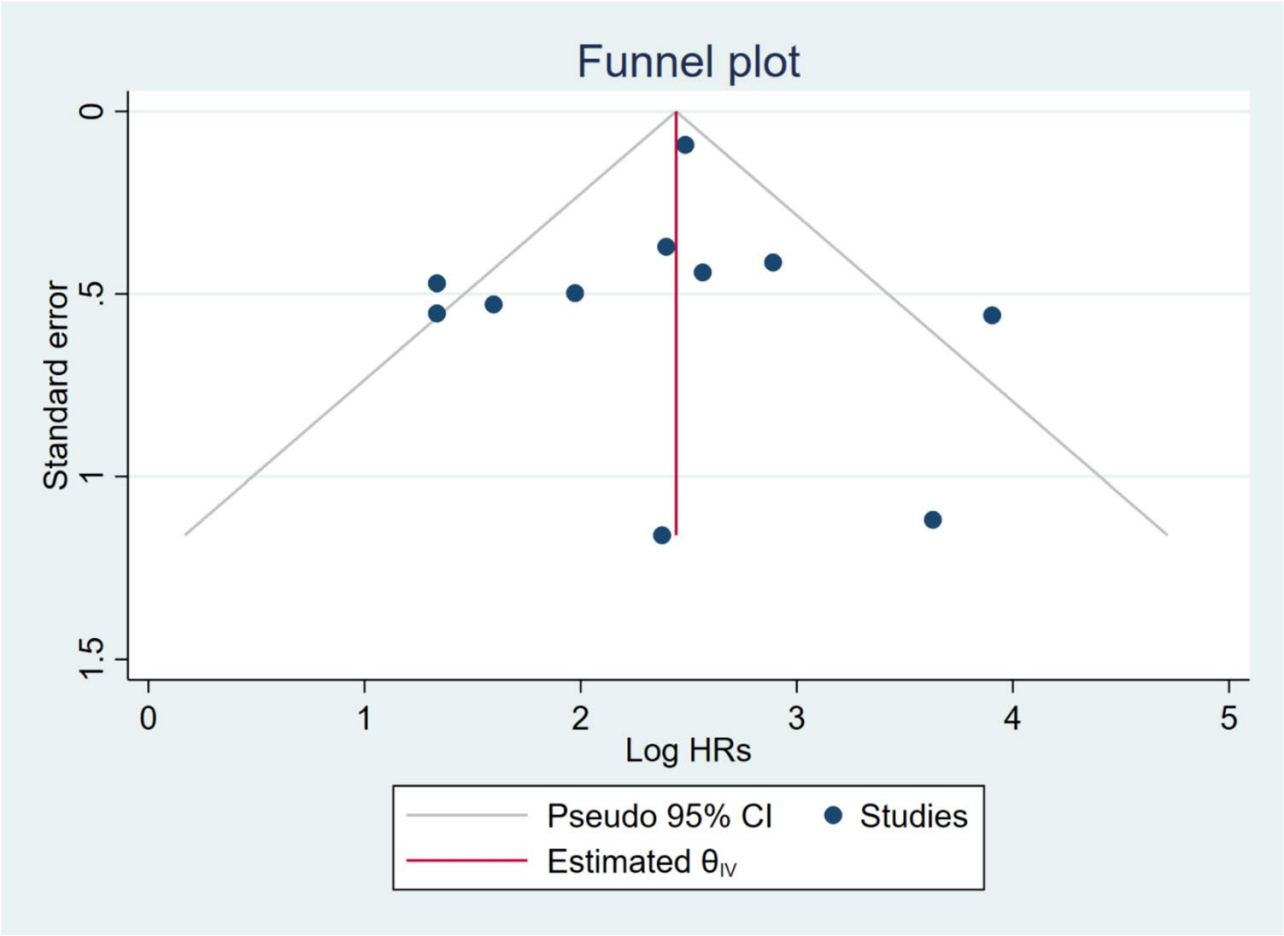
Funnel plot for assessing publication bias

Funnel plot analysis demonstrated no substantial evidence of publication bias (Fig. 8). Studies exhibited distribution around the pooled effect estimate with scatter patterns likely reflecting methodological diversity across investigations rather than systematic publication bias. The symmetrical distribution of studies surrounding the estimated effect magnitude substantiates the robustness of meta-analytic findings and suggests that unpublished studies are unlikely to substantially modify the conclusions. Egger's regression test revealed no evidence of publication bias (*β*₁ = 0.32, SE = 1.024, *z* = 0.31, *p* = 0.76), indicating that small-study effects did not significantly influence the pooled estimates.

**Fig. 9:**
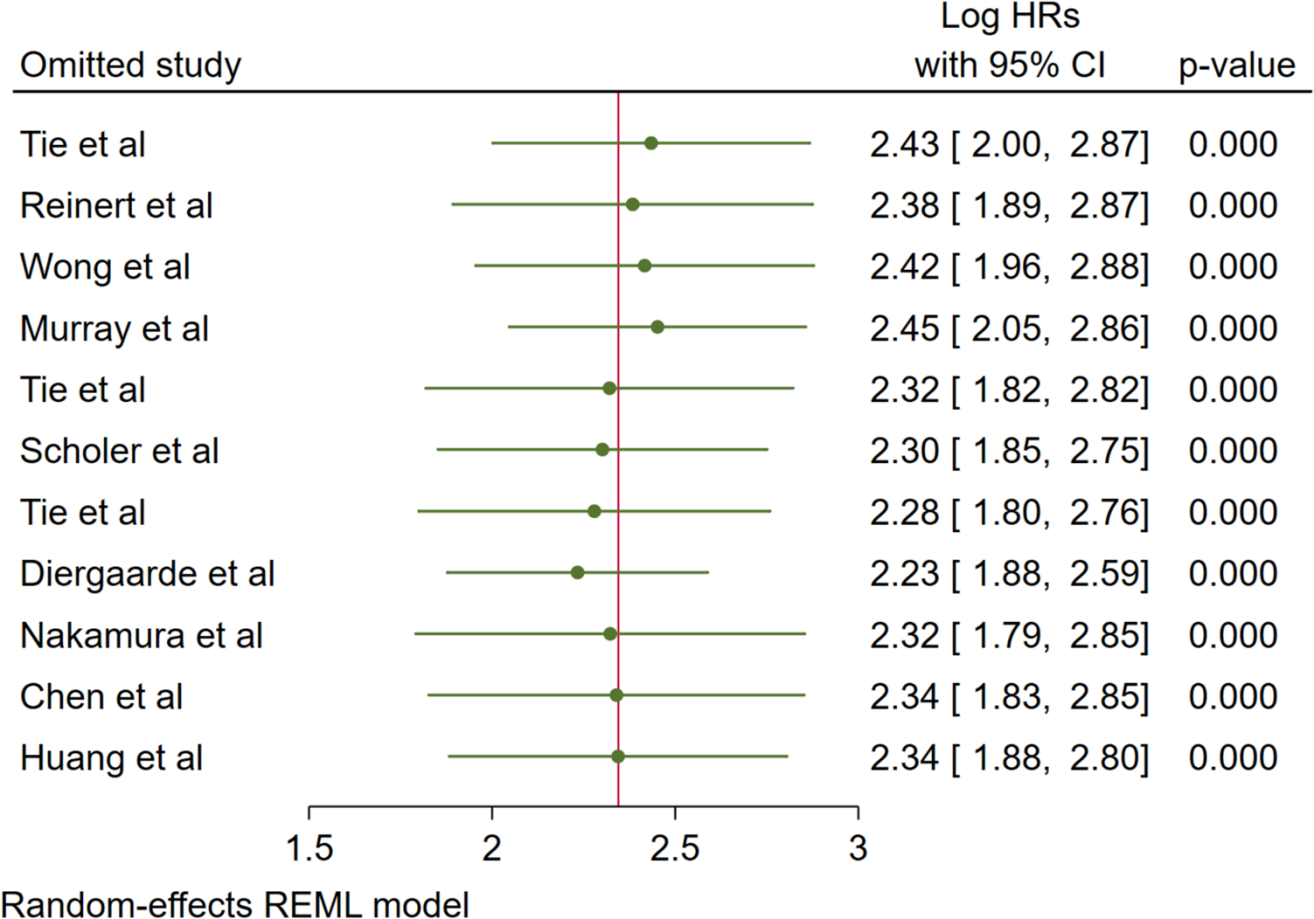
Sensitivity analysis

Leave-one-out sensitivity analysis was performed to assess the robustness of the pooled results by systematically excluding each study and recalculating the overall effect estimate. The analysis demonstrated remarkable stability of the meta-analytic findings, with log hazard ratios remaining consistently significant across all iterations (range: 2.23–2.45, all *p* < 0.001) (Fig. 9). The narrow range of effect estimates when individual studies were omitted indicates that no single study disproportionately influenced the overall results. This consistency across sensitivity analyses strengthens confidence in the reliability and robustness of the pooled effect estimate, confirming that the observed association is not driven by any particular study and supporting the validity of the meta-analytic conclusions.

## Discussion

This systematic review and meta-analysis provides comprehensive evidence substantiating the prognostic utility of circulating tumor DNA as a predictive biomarker for postsurgical colorectal cancer recurrence. Our findings demonstrate that patients harboring detectable ctDNA exhibit more than double the disease recurrence risk as compared to those with undetectable ctDNA, with a pooled hazard ratio of 2.34 (95% CI: 1.90–2.79).

### Clinical significance and superior performance over standard biomarkers

The clinical significance of these findings becomes particularly evident when contextualized against conventional surveillance markers currently employed in colorectal cancer management. Carcinoembryonic antigen (CEA), the only widely endorsed serum biomarker for postoperative monitoring, demonstrates limited sensitivity (40–70%) and specificity (80–90%) for detecting recurrence, often failing to identify early, treatable disease. Radiological surveillance, while highly specific, typically detects macroscopic disease and may miss minimal residual disease that ctDNA can identify at the molecular level. Furthermore, unlike histopathological staging which provides static prognostic information at diagnosis, ctDNA offers dynamic, real-time monitoring capability that can adapt to evolving tumor biology and treatment response.

In contrast, our meta-analysis demonstrates that ctDNA achieves superior prognostic performance with a pooled hazard ratio of 2.34, indicating more than double the recurrence risk stratification capability as compared to conventional markers. The ability of ctDNA to detect tumor-specific mutations in circulation provides a more sensitive and earlier indicator of disease recurrence through its liquid biopsy approach, enabling non-invasive tumor burden monitoring and potentially facilitating detection of molecular residual disease preceding radiological manifestation. This enhanced sensitivity could facilitate more timely therapeutic intervention and potentially superior outcomes through earlier initiation of salvage therapy or adjuvant treatment protocol modifications.

### Understanding sources of heterogeneity

The moderate heterogeneity observed in our meta-analysis (*I*^2^ = 66.40%) warrants careful consideration, as it reflects the inherent diversity in methodologies across the included studies. The primary contributor to this heterogeneity is patient stage distribution, with studies encompassing a wide range from early-stage (I–III) to advanced metastatic disease (I–IV). Our subgroup analysis by cancer stage demonstrated how this variability affects prognostic utility, with exceptional consistency observed in stage I–III patients (*I*^2^ = 0.00%). Particularly noteworthy is the substantial heterogeneity observed in the Stage III subgroup (*I*^2^ = 90.63%), which warrants specific consideration. This high variability likely reflects the inherent biological complexity of Stage III colorectal cancer, where nodal involvement patterns vary considerably—from minimal nodal disease (N1) to extensive regional lymph node involvement (N2). The heterogeneous tumor biology within Stage III disease, including differences in microsatellite instability status, tumor mutational burden, and vascular invasion patterns, may influence ctDNA shedding kinetics and detection rates. In addition, treatment heterogeneity within Stage III patients, particularly variations in adjuvant chemotherapy regimens and duration, could contribute to the observed variability in prognostic associations. These factors collectively underscore the complexity of Stage III disease and suggest that future research should stratify Stage III patients by more granular clinical and molecular characteristics (Online Resource 4).

Timing of ctDNA sampling represents another significant source of heterogeneity, with collection timepoints varying from immediate postoperative periods (3–7 days) to longer-term surveillance intervals (every 3 months). This temporal variability influences ctDNA detection rates and prognostic accuracy, as ctDNA levels fluctuate post-surgery and in response to adjuvant therapies. In addition, the diversity of ctDNA detection platforms contributes to methodological heterogeneity, with studies employing droplet digital PCR (ddPCR), next-generation sequencing (NGS), and Safe-SeqS technologies. Finally, the design of mutational panels varies considerably, ranging from single-gene assays (*KRAS*, *BRAF*) to comprehensive multi-gene panels, affecting the likelihood of detecting tumor-specific mutations.

Despite these sources of variability, the consistent direction of effect across all studies (HR > 1) supports the robustness of ctDNA as a prognostic biomarker, indicating that the observed heterogeneity reflects methodological diversity rather than fundamental inconsistencies in ctDNA's prognostic utility.

### Technology-agnostic performance across detection platforms

Our analysis reveals that ctDNA maintains consistent prognostic utility across different detection platforms, though important methodological distinctions merit consideration. Safe-SeqS (employed in 4 studies) offers high sensitivity for detecting low-frequency mutations but remains limited to predefined hotspot regions. Next-generation sequencing (utilized in 4 studies) provides broader genomic coverage and can detect novel mutations, though with potentially variable sensitivity depending on sequencing depth and bioinformatics pipelines. Droplet digital PCR (used in 1 study) delivers exceptional sensitivity for known mutations but is restricted to a limited number of targets per assay.

Despite these technical differences, our subgroup analysis revealed no statistically significant differences between platforms (*p* = 0.10), with hazard ratios ranging from 2.16 (Safe-SeqS) to 3.63 (ddPCR). This consistency across methodologies supports the technology–agnostic nature of ctDNA as a prognostic biomarker. The methodology-based analysis revealed intriguing performance patterns, with droplet digital PCR demonstrating substantial effect magnitude (HR: 3.63), suggesting superior analytical sensitivity for specific targeted mutations, while next-generation sequencing approaches exhibited robust and consistent performance (HR: 2.67) across diverse platforms and analytical pipelines.

### Stage-specific utility and treatment-related insights

The consistency of our findings across diverse cancer stages holds particular clinical relevance for treatment decision-making. The subgroup analysis of stage I–III patients demonstrated exceptional consistency (HR: 2.04, *I*^2^ = 0.00%), suggesting that ctDNA exhibits particular utility in early-stage disease where curative intent remains the primary therapeutic objective and where identifying high-risk recurrence patients could substantially influence adjuvant therapeutic decision-making.

Subgroup stratification by adjuvant chemotherapy status revealed important treatment-related insights that challenge the assumption of uniform prognostic utility. Patients without adjuvant chemotherapy showed a pooled HR of 1.70 (95% CI: 1.07–2.34) with no heterogeneity (*I*^2^ = 0.00%), while patients receiving adjuvant chemotherapy demonstrated a significantly higher HR of 2.50 (95% CI: 2.08–2.93) with moderate heterogeneity (*I*^2^ = 58.00%). The statistically significant subgroup difference (*p* = 0.04) indicates that ctDNA demonstrates stronger prognostic value in patients receiving adjuvant chemotherapy, potentially reflecting the ability of ctDNA to identify patients with chemotherapy-resistant minimal residual disease.

### Extended prognostic utility beyond recurrence

The prognostic utility of ctDNA extends beyond recurrence prediction to encompass broader survival outcomes, reinforcing its clinical value. Analysis of overall survival data from two studies demonstrated a pooled hazard ratio of 2.24 (95% CI: 1.83–2.65; *p* < 0.001), indicating that ctDNA-positive patients exhibited more than double the mortality risk compared to ctDNA-negative counterparts. The analysis showed perfect homogeneity (*I*^2^ = 0.00%), supporting consistent prognostic value across different patient populations.

Surveillance recurrence-free survival analysis of four studies demonstrated even more pronounced prognostic performance with a pooled hazard ratio of 3.54 (95% CI: 3.30–3.77; *p* < 0.001) and no heterogeneity (*I*^2^ = 0.00%). This exceptional consistency suggests robust prognostic utility across different surveillance protocols and reinforces the potential for ctDNA-guided surveillance strategies in clinical practice.

### Clinical implementation and pragmatic considerations

Although our findings robustly support ctDNA prognostic utility, successful clinical implementation requires addressing several pragmatic considerations. Standardization of ctDNA detection methodologies remains paramount, as different platforms exhibit varying analytical performance characteristics, detection limits, and specificity for diverse genetic alteration types. Standardized protocol development for sample collection, processing, storage, and analysis will prove essential for widespread clinical adoption and inter-laboratory reproducibility.

Cost-effectiveness considerations remain important for healthcare systems, as ctDNA testing currently exceeds conventional surveillance method costs. However, the potential for earlier recurrence detection, personalized treatment approaches, and unnecessary treatment avoidance may justify additional costs through improved patient outcomes and more efficient healthcare resource utilization. Quality assurance measures, including appropriate controls and standardized reporting formats, will be crucial for ensuring reliable results across diverse healthcare environments.

### Literature concordance and clinical validation

Our findings demonstrate concordance with previous systematic reviews examining ctDNA in colorectal malignancies, though our analysis incorporates more contemporary studies and focuses specifically on predictive utility for postsurgical recurrence. The hazard ratio of 2.34 observed in our analysis demonstrates comparability with previous reports, reinforcing ctDNA robustness as a prognostic biomarker across diverse study populations and temporal periods.

Clinical validation of ctDNA monitoring has been substantiated through several landmark prospective investigations, with recent randomized controlled trials demonstrating that ctDNA-guided therapeutic decisions can enhance patient outcomes compared to standard surveillance approaches. This provides proof-of-concept for ctDNA clinical utility beyond prognostic value and supports the transition from research applications to clinical implementation.

### Limitations and future research directions

Several limitations warrant acknowledgment in our analysis. The moderate inter-study heterogeneity, while systematically explored through subgroup analyses, reflects differences in patient populations, ctDNA detection methodologies, follow-up protocols, and study designs. The number of incorporated studies, while substantial for this emerging field, was constrained by relatively recent ctDNA technology development and requisite adequate follow-up periods for recurrence outcome assessment.

Most studies were conducted in developed nations with advanced healthcare systems and standardized surgical and oncological care protocols. Findings generalizability to alternative healthcare settings may be limited by differences in surgical techniques, adjuvant therapy protocols, follow-up practices, and patient populations with distinct genetic backgrounds or comorbidity profiles.

Future research should concentrate on large prospective randomized trials examining ctDNA-guided treatment decisions to definitively establish clinical utility beyond prognostic value and inform treatment guideline development. Technical advances in ctDNA detection, including enhanced analytical sensitivity, reduced costs, abbreviated turnaround times, and pan-cancer approach development, may further augment predictive value and practical utility of liquid biopsy monitoring.

## Conclusion

This systematic review and meta-analysis provides compelling evidence substantiating the prognostic utility of circulating tumor DNA for postsurgical colorectal cancer recurrence. The consistent and significant association between ctDNA positivity and augmented recurrence risk (HR: 2.34; 95% CI: 1.90–2.79) across multiple investigations, diverse patient populations, and various methodological approaches robustly supports ctDNA clinical utility as a predictive biomarker.

ctDNA monitoring implementation in clinical practice possesses the potential to revolutionize colorectal cancer surveillance by enabling more personalized monitoring strategies, facilitating earlier recurrence detection, and optimizing therapeutic decisions. The particularly robust and consistent results observed in early-stage (I-III) disease suggest that ctDNA monitoring may demonstrate especial value in patients where curative surgical intervention constitutes the primary therapeutic objective.

Future research should concentrate on standardizing detection methodologies, optimizing monitoring protocols, and conducting large prospective randomized trials to definitively establish ctDNA-guided treatment approach clinical utility. ctDNA monitoring integration into routine clinical practice represents a significant advancement toward precision oncology in colorectal cancer management, with the ultimate objective of enhancing long-term patient outcomes through more personalized and efficacious surveillance strategies.

## Supplementary Information

Below is the link to the electronic supplementary material.

Supplementary file1 (DOCX 26 KB)

Supplementary file2 (DOCX 269 KB)

## Data Availability

The data set used during the study are available from the corresponding author on a reasonable request.

## References

[1] Sung H, Ferlay J, Siegel RL, Laversanne M, Soerjomataram I, Jemal A, et al. Global cancer statistics 2020: GLOBOCAN estimates of incidence and mortality worldwide for 36 cancers in 185 countries. CA Cancer J Clin. 2021;71:209–49. 33538338 10.3322/caac.21660

[2] Bray F, Laversanne M, Sung H, Ferlay J, Siegel RL, Soerjomataram I, et al. Global cancer statistics 2022: GLOBOCAN estimates of incidence and mortality worldwide for 36 cancers in 185 countries. CA Cancer J Clin. 2024;74:229–63. 38572751 10.3322/caac.21834

[3] Ryuk JP, Choi GS, Park JS, Kim HJ, Park SY, Yoon GS, et al. Predictive factors and the prognosis of recurrence of colorectal cancer within 2 years after curative resection. Ann Surg Treat Res. 2014;86:143–51. 24761423 10.4174/astr.2014.86.3.143 PMC3994626

[4] Renehan AG, Egger M, Saunders MP, O’Dwyer ST. Impact on survival of intensive follow up after curative resection for colorectal cancer: systematic review and meta-analysis of randomised trials. BMJ. 2002;324:813. 11934773 10.1136/bmj.324.7341.813 PMC100789

[5] Van Der Stok EP, Spaander MCW, Grünhagen DJ, Verhoef C, Kuipers EJ. Surveillance after curative treatment for colorectal cancer. Nat Rev Clin Oncol. 2016;14(5):297–315. 27995949 10.1038/nrclinonc.2016.199

[6] O’Connell JB, Maggard MA, Ko CY. Colon cancer survival rates with the new American joint committee on cancer sixth edition staging. JNCI J Natl Cancer Inst. 2004;96:1420–5. 15467030 10.1093/jnci/djh275

[7] Li J, Guo BC, Sun LR, Wang JW, Fu XH, Zhang SZ, et al. TNM staging of colorectal cancer should be reconsidered by T stage weighting. World J Gastroenterol. 2014;20:5104–12. 24803826 10.3748/wjg.v20.i17.5104 PMC4009548

[8] Greene FL, Stewart AK, Norton HJ, Cohen AM. A new TNM staging strategy for node-positive (stage III) colon cancer: an analysis of 50,042 patients. Ann Surg. 2002;236:416. 12368669 10.1097/00000658-200210000-00003 PMC1422595

[9] Nitsche U, Maak M, Schuster T, Künzli B, Langer R, Slotta-Huspenina J, et al. Prediction of prognosis is not improved by the seventh and latest edition of the TNM classification for colorectal cancer in a single-center collective. Ann Surg. 2011;254:793–800. 22042471 10.1097/SLA.0b013e3182369101

[10] Gooiker GA, Dekker JWT, Bastiaannet E, Van Der Geest LGM, Merkus JWS, Van De Velde CJH, et al. Risk factors for excess mortality in the first year after curative surgery for colorectal cancer. Ann Surg Oncol. 2012;19:2428–34. 22396000 10.1245/s10434-012-2294-6 PMC3404283

[11] Duffy MJ. Carcinoembryonic antigen as a marker for colorectal cancer: is it clinically useful? Clin Chem. 2001;47:624–30. 11274010

[12] Wang R, Wang Q, Li P. Significance of carcinoembryonic antigen detection in the early diagnosis of colorectal cancer: a systematic review and meta-analysis. World J Gastrointest Surg. 2023;15:2907–18. 38222002 10.4240/wjgs.v15.i12.2907 PMC10784816

[13] Shinkins B, Nicholson BD, James T, Pathiraja I, Pugh S, Perera R, et al. What carcinoembryonic antigen level should trigger further investigation during colorectal cancer follow-up? a systematic review and secondary analysis of a randomised controlled trial. Health Technol Assess. 2017;21:1. 28617240 10.3310/hta21220 PMC5483644

[14] Fakih M, Sandhu J, Wang C, Kim J, Chen YJ, Lai L, et al. Evaluation of comparative surveillance strategies of circulating tumor DNA, imaging, and carcinoembryonic antigen levels in patients with resected colorectal cancer. JAMA Netw Open. 2022;5:e221093–e221093. 35258578 10.1001/jamanetworkopen.2022.1093 PMC8905389

[15] Chin RI, Chen K, Usmani A, Chua C, Harris PK, Binkley MS, et al. Detection of solid tumor molecular residual disease (MRD) using circulating tumor DNA (ctDNA). Mol Diagn Ther. 2019;23(3):311–31. 30941670 10.1007/s40291-019-00390-5 PMC6561896

[16] Moding EJ, Nabet BY, Alizadeh AA, Diehn M. Detecting liquid remnants of solid tumors: circulating tumor DNA minimal residual disease. Cancer Discov. 2021;11:2968–86. 34785539 10.1158/2159-8290.CD-21-0634 PMC8976700

[17] Pantel K, Alix-Panabières C. Liquid biopsy and minimal residual disease — latest advances and implications for cure. Nat Rev Clin Oncol. 2019;16(7):409–24. 30796368 10.1038/s41571-019-0187-3

[18] Chen H, Zhou Q. Detecting liquid remnants of solid tumors treated with curative intent: circulating tumor DNA as a biomarker of minimal residual disease (review). Oncol Rep. 2023;49:106. 37052271 10.3892/or.2023.8543 PMC10152452

[19] Zhu L, Xu R, Yang L, Shi W, Zhang Y, Liu J, et al. Minimal residual disease (MRD) detection in solid tumors using circulating tumor DNA: a systematic review. Front Genet. 2023;14:1172108. 37636270 10.3389/fgene.2023.1172108 PMC10448395

[20] Semenkovich NP, Szymanski JJ, Earland N, Chauhan PS, Pellini B, Chaudhuri AA. Genomic approaches to cancer and minimal residual disease detection using circulating tumor DNA. J Immunother Cancer. 2023;11:6284. 10.1136/jitc-2022-006284 PMC10314661 37349125

[21] Galvano A, Taverna S, Badalamenti G, Incorvaia L, Castiglia M, Barraco N, et al. Detection of RAS mutations in circulating tumor DNA: a new weapon in an old war against colorectal cancer a systematic review of literature and meta-analysis. Ther Adv Med Oncol. 2019;11:1758835919874653. 31534493 10.1177/1758835919874653 PMC6737868

[22] Nadal C, Winder T, Gerger A, Tougeron D. Future perspectives of circulating tumor DNA in colorectal cancer. Tumour Biol. 2017. 10.1177/1010428317705749. 28488528 10.1177/1010428317705749

[23] Page MJ, McKenzie JE, Bossuyt PM, Boutron I, Hoffmann TC, Mulrow CD, et al. The PRISMA 2020 statement: an updated guideline for reporting systematic reviews. BMJ. 2021. 10.1136/BMJ.N71. 33782057 10.1136/bmj.n71 PMC8005924

[24] Stang A. Critical evaluation of the Newcastle-ottawa scale for the assessment of the quality of nonrandomized studies in meta-analyses. Eur J Epidemiol. 2010;25:603–5. 20652370 10.1007/s10654-010-9491-z

[25] Wells G, Shea B, O’Connell D, Peterson J, Welch V, Losos M, Tugwell P. Ottawa Hospital Research Institute. In: The Newcastle-Ottawa Scale (NOS) for assessing the quality of nonrandomised studies in meta-analyses (2012). https://www.ohri.ca/programs/clinical_epidemiology/oxford.asp. Accessed 30 Aug 2025

[26] DerSimonian R, Laird N. Meta-analysis in clinical trials. Control Clin Trials. 1986;7:177–88. 3802833 10.1016/0197-2456(86)90046-2

[27] Higgins JPT, Thompson SG, Deeks JJ, Altman DG. Measuring inconsistency in meta-analyses. BMJ. 2003;327:557–60. 12958120 10.1136/bmj.327.7414.557 PMC192859

[28] Egger M, Smith GD, Schneider M, Minder C. Bias in meta-analysis detected by a simple, graphical test. BMJ. 1997;315:629–34. 9310563 10.1136/bmj.315.7109.629 PMC2127453

[29] Tie J, Cohen JD, Wang Y, Christie M, Simons K, Lee M, et al. Circulating tumor DNA analyses as markers of recurrence risk and benefit of adjuvant therapy for stage III colon cancer. JAMA Oncol. 2019;5:1710–7. 31621801 10.1001/jamaoncol.2019.3616 PMC6802034

[30] Reinert T, Henriksen TV, Christensen E, Sharma S, Salari R, Sethi H, et al. Analysis of plasma cell-free DNA by ultradeep sequencing in patients with stages I to III colorectal cancer. JAMA Oncol. 2019;5:1124–31. 31070691 10.1001/jamaoncol.2019.0528 PMC6512280

[31] Wong R, Tie J, Lee M, Cohen J, Wang Y, Li L, et al. The potential role of circulating tumor DNA (ctDNA) in the further investigation of colorectal cancer patients with nonspecific findings on standard investigations. Int J Cancer. 2019;145:540–7. 30628066 10.1002/ijc.32117 PMC6563608

[32] Murray DH, Symonds EL, Young GP, Byrne S, Rabbitt P, Roy A, et al. Relationship between post-surgery detection of methylated circulating tumor DNA with risk of residual disease and recurrence-free survival. J Cancer Res Clin Oncol. 2018;144:1741–50. 29992492 10.1007/s00432-018-2701-x PMC11813478

[33] Tie J, Cohen JD, Wang Y, Li L, Christie M, Simons K, et al. Serial circulating tumour DNA analysis during multimodality treatment of locally advanced rectal cancer: a prospective biomarker study. Gut. 2019;68(4):663–71. 29420226 10.1136/gutjnl-2017-315852 PMC6265124

[34] Schøler LV, Reinert T, Ørntoft MBW, Kassentoft CG, Árnadóttir SS, Vang S, et al. Clinical implications of monitoring circulating tumor DNA in patients with colorectal cancer. Clin Cancer Res. 2017;23:5437–45. 28600478 10.1158/1078-0432.CCR-17-0510

[35] Tie J, Wang Y, Tomasetti C, Li L, Springer S, Kinde I, et al. Circulating tumor DNA analysis detects minimal residual disease and predicts recurrence in patients with stage II colon cancer. Sci Transl Med. 2016. 10.1126/SCITRANSLMED.AAF6219/SUPPL_FILE/8-346RA92_SM.PDF. 27384348 10.1126/scitranslmed.aaf6219 PMC5346159

[36] Diergaarde B, Young G, Hall DW, Mazloom A, Costa GL, Subramaniam S, et al. Circulating tumor DNA as a marker of recurrence risk in stage III colorectal cancer: the α-CORRECT study. J Surg Oncol. 2025;132:175–86. 39865324 10.1002/jso.27989 PMC12302971

[37] Chen G, Peng J, Xiao Q, Wu HX, Wu X, Wang F, et al. Postoperative circulating tumor DNA as markers of recurrence risk in stages II to III colorectal cancer. J Hematol Oncol. 2021;14:1–11. 34001194 10.1186/s13045-021-01089-z PMC8130394

[38] Nakamura Y, Watanabe J, Akazawa N, Hirata K, Kataoka K, Yokota M, et al. ctDNA-based molecular residual disease and survival in resectable colorectal cancer. Nat Med. 2024;30:3272–83. 39284954 10.1038/s41591-024-03254-6 PMC11564113

[39] Huang K, Qu H, Zhang X, Huang T, Sun X, He W, et al. Circulating tumor DNA sequencing for colorectal cancers: A comparative analysis of colon cancer and rectal cancer data. Cancer Biomark. 2019;26:313–22. 31561327 10.3233/CBM-190257 PMC12826425

