# Supplementary material for: Circulating tumor DNA as a predictive biomarker for colorectal cancer postsurgical recurrence: a systematic review and meta-analysis
Source: Clin Transl Oncol. 2025 Oct 17;28(4):1348–62. doi: 10.1007/s12094-025-04073-y (PMC13009131; doi:10.1007/s12094-025-04073-y)
Supplement: Supplementary file 1 — Supplementary file1 (DOCX 26 KB) [file 12094_2025_4073_MOESM1_ESM.docx]

**Supplementary Information**

**Circulating Tumor DNA as a Predictive Biomarker for Colorectal Cancer Postsurgical Recurrence: A Systematic Review and Meta-Analysis**

Online Resource 1. Detailed search strategy

| **Database** | **Keywords** | **Results** |
| --- | --- | --- |
| PubMed | (prognosis OR predictive OR prediction OR biomarker) | 5,135,237 |
|  | ("colorectal cancer" OR "colon cancer" OR "rectal cancer" OR CRC) | 249,202 |
|  | ("circulating tumor DNA" OR ctDNA OR "circulating tumour DNA" OR "cell-free DNA" OR cfDNA) | 22,559 |
|  | (recurrence OR "disease recurrence" OR "cancer relapse" OR "tumor relapse" OR "recurrence-free survival" OR RFS) | 927,098 |
|  | #1 AND #2 AND #3 AND #4 | 355 |
| Web of Science | colorectal cancer OR colon cancer OR rectal cancer OR CRC AND circulating tumor DNA OR ctDNA OR circulating tumour DNA OR cell-free DNA OR cfDNA AND recurrence OR disease recurrence OR cancer relapse OR tumor relapse OR recurrence-free survival OR RFS AND prognosis OR predictive OR prediction OR biomarker | 387 |
| Embase | colorectal cancer OR colon cancer OR rectal cancer OR CRC AND circulating tumor DNA OR ctDNA OR circulating tumour DNA OR cell-free DNA OR cfDNA AND recurrence OR disease recurrence OR cancer relapse OR tumor relapse OR recurrence-free survival OR RFS AND prognosis OR predictive OR prediction OR biomarker | 487 |
| Scopus | colorectal cancer OR colon cancer OR rectal cancer OR CRC AND circulating tumor DNA OR ctDNA OR circulating tumour DNA OR cell-free DNA OR cfDNA AND recurrence OR disease recurrence OR cancer relapse OR tumor relapse OR recurrence-free survival OR RFS AND prognosis OR predictive OR prediction OR biomarker | 980 |

Online Resource 2. Baseline characteristics

| **Study ID** | **Year** | **Setting** | **Type of cancer** | **Stage of cancer** | **Detection Assay** | **Lead Time (months)** | **Adjuvant Chemotherapy** | **Median follow-up*** | **N total** | **R (n/N) +** | **R (n/N) -** | **Median RFS (+)*** | **HR** |
| --- | --- | --- | --- | --- | --- | --- | --- | --- | --- | --- | --- | --- | --- |
| Tie et al | 2019 | Australia | Colon cancer | Stage III | Safe-SeqS | NR | Y | 28.9 | 96 | 10/20 | 14/76 | 20.63 | 3.8 |
| Reinert et al | 2019 | Denmark | CRC | Stage I–III | NGS | 16.5 m | Y | 12.5 | 94 | 7/10 | 10/84 | 12.13 | 7.2 |
| Wong et al | 2019 | Australia | CRC | Stage I–IV | Safe-SeqS | NR | Y | 24 | 43 | 11/14 | 10/29 | 3.46 | 4.94 |
| Murray et al | 2018 | German | CRC | Stage I–IV | qPCR | NR | N | 22.9 | 172 | 7/28 | 16/144 | 33.62 | 3.8 |
| Tie et al | 2019 | Australia | Rectal cancer | Stage II/III | Safe-SeqS | NR | Y | 24 | 159 | 11/19 | 12/140 | 11.36 | 13 |
| Scholer et al | 2017 | Denmark | CRC | Stage I–IV | ddPCR | 9.4 m | Y | NR | 27 | 14/14 | 0/13 | 9.42 | 37.7 |
| Tie et al | 2016 | Australia | Colon cancer | Stage II | Safe-SeqS | 5.5 m | N | 27 | 230 | 11/20 | 16/210 | 7.42 | 18 |
| Diergaarde et al | 2025 | USA | CRC | Stage III | NGS | 10.4 m | Y | 57.6 | 124 | 20/27 | 2/84 | NR | 49.6 |
| Nakamura et al | 2024 | Japan/Taiwan | CRC | Stage I-IV | NR | 5.91 m | Y | 23 | 2,109 | 263/336 | 233/1773 | 5.34 | 11.99 |
| Chen et al | 2021 | China | CRC | Stage II/III | NGS | 5.01 m | Y | 27.4 | 240 | 12/20 | 24/220 | NR | 10.98 |
| Huang et al | 2019 | China | CRC | Stage I–III | NGS | 10.5 m | Y | 30 | 39 | 3/9 | 1/30 | NR | 10.77 |

Online Resource 3. Study quality assessment utilizing (NOS) scale.

| **Study** | **Year** | **Selection** | | | | **Comparability** | **Outcome** | | | **Total score (out of 9)** |
| --- | --- | --- | --- | --- | --- | --- | --- | --- | --- | --- |
|  |  | **Adequate definition of patient cases (Max 1)** | **Representativeness of patient cases (Max 1)** | **Selection of Controls (Max 1)** | **Definition of Controls (Max 1)** | **Control for important or additional factors (Max 2)** | **Ascertainment of exposure (Max 1)** | **Was follow up long enough for outcomes to occur? (Max 1)** | **Adequacy of follow up (Max 1)** |  |
| Tie et al | 2019 | 1 | 1 | 1 |  | 2 | 1 | 1 | 1 | 8 |
| Reinert et al | 2019 | 1 | 1 | 1 |  | 1 |  | 1 | 1 | 6 |
| Wong et al | 2019 | 1 | 1 | 1 | 1 | 2 | 1 | 1 | 1 | 9 |
| Murray et al | 2018 | 1 | 1 |  |  | 2 | 1 | 1 | 1 | 7 |
| Tie et al | 2019 | 1 | 1 | 1 |  | 2 | 1 | 1 | 1 | 8 |
| Scholer et al | 2017 | 1 | 1 | 1 | 1 | 2 | 1 | 1 | 1 | 8 |
| Tie et al | 2016 | 1 | 1 | 1 |  | 2 | 1 | 1 | 1 | 8 |
| Diergaarde et al | 2025 | 1 | 1 | 1 |  | 1 | 1 | 1 | 1 | 7 |
| Nakamura et al | 2024 | 1 | 1 | 1 |  | 2 | 1 | 1 | 1 | 8 |
| Chen et al | 2021 | 1 | 1 | 1 |  | 1 | 1 | 1 | 1 | 7 |
| Huang et al | 2019 | 1 | 1 | 1 |  | 2 | 1 | 1 | 1 | 8 |

Online Resource 4. Potential Sources of Heterogeneity

| **Study ID** | **Year** | **Mutation Panel for ctDNA detection (Recurrent)** | **Sample size by cancer stage I/II/III)** | **Standard** | **Detection Assay** | **ctDNA Timepoint Post Surgery** | **Other Endpoints** | **HRs** | **ACT** | **ACT vs RFS(p value)** | **Lead Time (months)** |
| --- | --- | --- | --- | --- | --- | --- | --- | --- | --- | --- | --- |
| Tie et al | 2019 | APC, BRAF, TP53, KRAS, NRAS, RNF | 96 (Stage III) | radiological diagnosis | Safe-SeqS | 4 to 10 weeks PS | NR |  | Y | 6.8 (< .001) | NR |
| Reinert et al | 2019 | KRAS, PIK3CA, SMAD4, BRAF | 5/38/79 | standard-of-care radiologic imaging | NGS | 30th day and every 3rd month PS | Surveilance RFS | 43.5 | Y | 17.5 (< .001) | 16.5 m |
| Wong et al | 2019 | NR | 4/18/2020 | histological/radiological diagnosis | Safe-SeqS | within 10 weeks PS | NR |  | N | NR | NR |
| Murray et al | 2018 | BCAT1 and IKZF1 | NR | radiological imaging | qPCR | within 12 months PS | NR |  | N | NR | NR |
| Tie et al | 2019 | NR | Stage II: 35, Stage III: 124 | MRI | Safe-SeqS | 4 to 10 weeks PS | NR |  | Y | 10 (<.001) | NR |
| Scholer et al | 2017 | KRAS, BRAF | NR | Histological diagnosis | ddPCR | 8, 30, and every 3 months PS | OS | 6.7 | N | NR | 9.4 m |
| Tie et al | 2016 | PIK3CA, NRAS, KRAS, POLE, SMAD4, TP53, APC, FBXW7 | Staage II: 230 | radiological imaging | Safe-SeqS | 4 to 10 weeks PS | NR |  | Y | 11 (0.001) | 5.5 m |
| Diergaarde et al | 2025 | NR | 124 (Stage III) | radiological imaging | NGS | 3–12 weeks PS | ACT and Surveilance RFS | 49.6 | Y | 16.7 (< 0.0001) | 10.4 m |
| Nakamura et al | 2024 | NR | 234/632/936/418 | histological/radiological diagnosis | NR | 4, 12, 24, 36, 48, 72 and 96 weeks PS | Surveilance RFS, ACT and OS | Surveilance RFS: 33.56, OS: 9.68 | Y | 0.23 (< 0.0001) | 5.91 m |
| Chen et al | 2021 | SMAD4, PTEN and PKHD1 | Stage II: 112, Stage III: 128 | radiological imaging | NGS | 3–7 days and 6 month PS | Surveilance RFS, ACT | Surveilance RFS: 32.02 | Y | 12.76 (<.001) | 5.01 m |
| Huang et al | 2019 | APC, TP53, KRAS, PIK3CA and BRAF | 11/13/11/4 | radiological imaging | NGS | 1 month, two months and half a year PS | NR |  | N | NR | 10.5 m |
